# (–)–Epicatechin combined with 8 weeks of treadmill exercise is associated with increased angiogenic and mitochondrial signaling in mice

**DOI:** 10.3389/fphar.2015.00043

**Published:** 2015-03-13

**Authors:** Icksoo Lee, Maik Hüttemann, Adele Kruger, Aliccia Bollig-Fischer, Moh H. Malek

**Affiliations:** ^1^College of Medicine, Dankook University, Cheonan-si, South Korea; ^2^Center for Molecular Medicine and Genetics, Wayne State University School of Medicine, Detroit, MI, USA; ^3^Cardiovascular Research Institute, Wayne State University School of Medicine, Detroit, MI, USA; ^4^Department of Obstetrics and Gynecology, Wayne State University, Detroit, MI, USA; ^5^Department of Oncology, Wayne State University, Detroit, MI, USA; ^6^Integrative Physiology of Exercise Laboratory, Department of Health Care Sciences, Wayne State University Eugene Applebaum College of Pharmacy and Health Sciences, Detroit, MI, USA

**Keywords:** exercise tolerance, muscle, skeletal, fatigue, mouse model, mitochondrial proteins

## Abstract

The purpose of this study was to conduct an 8 week endurance training program with and without (–)–epicatechin treatment and to determine whether there is a possible cumulative effect on protein markers of angiogenesis and mitochondrial biogenesis. Thirty-four 14-month old male mice (C57BL/6N) were randomized into four groups: control (C); (–)–epicatechin only ((–)–Epi); control with endurance training (CE); and (–)–epicatechin with endurance training ((–)–Epi-Ex). Mice in the training groups performed treadmill exercise for 8 weeks (5 × /week for 60 min/session), whereas mice in the (–)–epicatechin group received 1.0 mg/kg of body mass twice daily during the training period. At 8 weeks, distance ran on the treadmill increased by 46, 69, and 84% in the (–)–Epi, CE, and (–)–Epi-Ex groups, respectively compared to the control group (*p* < 0.001 for all comparisons). Furthermore, the (–)–Epi-Ex group had significantly higher exercise capacity than the (–)–Epi and CE group. For angiogenic regulators, the (–)–Epi-Ex group had significantly higher VEGF-R2 protein expression with a concomitant reduction in TSP-1 protein expression than the exercise group. Interestingly, FoxO1 protein expression was significantly reduced for all three experimental groups compared to the control group. Protein markers such as PGC-1β and TFAM were significantly higher in the (–)–Epi-Ex group compared to the three other groups. These findings suggest that (–)–epicatechin treatment combined with 8 weeks of endurance training provide a cumulative effect on a number of angiogenic and mitochondrial signaling which functionally translates to enhanced exercise tolerance.

## INTRODUCTION

One of the foundations of exercise physiology is that endurance training increases the delivery and utilization of oxygen to the working muscle (Wagner, 1996; Saltin and Calbet, 2006). The delivery of oxygen from the blood to the muscle is facilitated by capillaries (Wagner, 1996). Studies have suggested that capillary growth results from a balance between pro-angiogenic factors such as vascular endothelial growth factor (VEGF-A; Olfert et al., 2009; Hüttemann et al., 2012; Lee et al., 2012; Malek et al., 2013) and anti-angiogenic factors such as thrombospondin-1 (TSP-1; Audet et al., 2011; Hüttemann et al., 2012; Roudier et al., 2012; Malek et al., 2013). Recently, Roudier et al. (2013) indicated that forkhead box O1 (FoxO1) deletion attenuated TSP-1 protein which improved blood flow in ischemic skeletal muscle of mice. Once the oxygenated blood is delivered to the muscle it is shuttled to the mitochondria (Wagner, 1996).

The ultimate goal of oxygen delivery and utilization is energy production in the mitochondria. This takes place in the electron transport chain, where cytochrome *c* oxidase (CcO) accepts electrons from cytochrome *c*, reduces 90% of cellular oxygen to water, and pumps protons across the inner mitochondrial membrane generating the mitochondrial membrane potential, which is used by ATP synthase to generate ATP from ADP and phosphate (Hüttemann et al., 2007). It has been suggested that CcO is the rate-limiting enzyme of the electron transport chain in mammals (Villani and Attardi, 1997; Villani et al., 1998; Acin-Perez et al., 2003; Piccoli et al., 2006; Dalmonte et al., 2009; Pacelli et al., 2011). Moreover, for robust aerobic energy production mitochondrial maintenance and biogenesis are crucial, and studies have shown that PGC-1 (peroxisome proliferator-activated receptor gamma coactivator 1) may play a role in this process (Pogozelski et al., 2009; Matiello et al., 2010; Rowe et al., 2011).

Previously, Nogueira et al. (2011) examined the effects of 15 days of (–)–epicatechin treatment with and without exercise on hindlimb skeletal muscle capillarity and mitochondrial biogenesis of 12 month old male mice. The investigators (Nogueira et al., 2011) stated, “…the intent was not to provide a training stimulus, but rather to determine if (–)–epicatechin needed a metabolic stimulus to have an effect, as reported for GW1516 by Narkar et al. (2008, p. 4617).” The investigators reported increased skeletal muscle capillarity, but did not examine protein expression of various angiogenic regulators. Nogueira et al. (2011), however, found increased protein expression for various mitochondrial complexes which corresponded to increases in mitochondrial volume and cristae abundance in the group receiving (–)–epicatechin compared to the vehicle group independent of the exercise intervention. The Nogueira et al. (2011) study provided initial data regarding the short-term effects of (–)–epicatechin treatment on skeletal muscle structure and function. Subsequent studies on (–)–epicatechin have shown that (–)–epicatechin attenuates detraining effects on skeletal muscle (Hüttemann et al., 2012) as well as increases angiogenic and mitochondrial signaling in rats with innate low running capacity (LCR; Hüttemann et al., 2013). These studies used either 14 (Hüttemann et al., 2012) or 30 days (Hüttemann et al., 2013) of (–)–epicatechin treatment in the absence of an endurance training intervention. These findings suggest, therefore, that (–)–epicatechin may function as a partial exercise mimetic. Nevertheless, a couple of critical questions about (–)–epicatechin treatment have yet to be answered: “*What are the long-term effects of* (–)–*epicatechin treatment on skeletal muscle function?*;” and “*Does* (–)–*epicatechin treatment in combination with endurance training provide a cumulative effect on delivery and utilization of oxygen in the hindlimb muscles?*”

The purpose of this study, therefore, was to conduct an 8 week endurance training program with and without (–)–epicatechin treatment and to determine whether there is a possible cumulative effect on protein markers of angiogenesis and mitochondrial biogenesis. We hypothesized that animals treated with (–)–epicatechin would have similar skeletal muscle adaptations as endurance trained animals. Furthermore, we hypothesized that the combination of (–)–epicatechin and endurance training would provide a cumulative effect for both angiogenic and mitochondrial signaling.

## MATERIALS METHODS

### ANIMAL APPROVAL AND HOUSING

Mice were 14-month old, male C57BL/6N (*N* = 34, Harlan Laboratories, Inc.) at the beginning and 16-month old at the termination of the study. We selected an older age relative to our initial study [12 month old; (Nogueira et al., 2011)] because Frazier et al. (2011) reported decreased mitochondrial function and endurance capacity with age. Animals were placed two per cage and provided a standard chow diet (Picolab® Laboratory Rodent Diet 5L0D; LabDiet, St. Louis, MO, USA) without restrictions. In addition, room temperature was maintained at 21 °C with 12-h light:dark cycles. All animal care and experimental procedures were approved by the Wayne State University Institutional Animal Care and Use Committee.

### RESEARCH DESIGN

A between-subjects design was used in which mice were randomized into four groups: (1) control (C); (2) (–)–epicatechin only ((–)–Epi); (3) control with endurance training (CE); and (4) (–)–epicatechin with endurance training ((–)–Epi-Ex). Thereafter, all groups performed a maximal treadmill test to exhaustion. A final maximal treadmill test was performed at 8 weeks.

### MAXIMAL TREADMILL TEST

All animals were familiarized with the treadmill the week prior to the maximal treadmill test. The mice ran on the treadmill (1055MSD Exer-6M, Columbus, OH, USA) at a slow speed (≈≈5 m min^–1^) at 0° incline for approximately 5–10 min. On the test day, animals began with a warm-up at 4 m min^–1^ for 2 min followed by an increase of 2 m min^–1^ every min thereafter. Testing of animals and determination of exhaustion was consistent with our previous rodent model studies (Malek and Olfert, 2009; Malek et al., 2010, 2013; Nogueira et al., 2011; Hüttemann et al., 2012; Lee et al., 2012). We used the total distance ran as the outcome variable for exercise tolerance because Koch and Britton (2001) as well as Girard et al. (2001) have shown this index to be the best predictor of endurance capacity.

### ENDURANCE TRAINING PROTOCOL FOR 8 WEEKS

For 8 weeks, animals in the CE and (–)–Epi-Ex groups performed treadmill exercise at 60% of the maximum work rate at 5° incline for 60 min 5 times per week (Monday through Friday). The training sessions were performed between 8:00 and 11:00 am. Variations of this protocol have shown increased angiogenic and mitochondrial adaptations in the working muscle (Hüttemann et al., 2012; Malek et al., 2013). Animals in the control and (–)–Epi groups were placed on a non-moving treadmill belt during each session.

### (–)–EPICATECHIN TREATMENT

Consistent with our previous work on (–)–epicatechin in mice (Nogueira et al., 2011; Hüttemann et al., 2012) and rats (Hüttemann et al., 2013) animals in the (–)–epicatechin groups were given a dosage of 1.0 mg/kg of body mass twice a day [morning (7:00 am) and evening (7:00 pm)]. Animals in groups 1 and 3 received vehicle (water). In our previous studies (Nogueira et al., 2011; Hüttemann et al., 2012) animals were given (–)–epicatechin (dissolved in water) on consecutive days during the treatment period. Recently, however, Hüttemann et al. (2013) showed sustained angiogenic and mitochondrial signaling 15 days after cessation of (–)–epicatechin treatment. Therefore, in the current study, animals were given either the vehicle or (–)–epicatechin (Sigma-Aldrich, St. Louis, MO, USA) using a Monday through Friday schedule for 8 weeks which would correspond with the exercise training regimen. Delivery of (–)–epicatechin and vehicle in the present study was via oral gavage by experienced personnel. The total volume of oral gavage per session per animal was ≈0.20 ml twice daily.

### TISSUE HARVESTING PROCEDURE

All animals were overdosed with sodium pentobarbital (200 mg/kg, *i.p.*) 72 h after the final maximal treadmill test. Thereafter the hindlimb muscles (quadriceps femoris, lateral gastrocnemius, and plantaris muscles) were harvested. This approach was taken in order to examine the basal protein expression in the muscle rather than examining the response immediately following exercise. The method of sectioning and storing the muscle was consistent with our previous work (Malek and Olfert, 2009; Malek et al., 2010, 2013; Nogueira et al., 2011; Hüttemann et al., 2012, 2013; Lee et al., 2012).

### MEASUREMENT OF CAPILLARITY

The Rosenblatt et al. (1987) capillary staining method was used for the plantaris muscle. Muscle sections were viewed under a digital microscope (20 × magnification, Leica DMD108, Buffalo Grove, IL, USA) and randomly selected for animals in each group corresponding to ≈85% of the entire muscle being analyzed for each animal. Quantification of capillaries were performed using the recommended method of Hepple (Hepple, 1997; Hepple and Mathieu-Costello, 2001) by measuring the following: (1) the number of capillaries around a fiber (N*_CAF_*), (2) the capillary-to-fiber ratio on an individual-fiber basis (C/F*_i_*), and (3) capillary density (CD) which was calculated by using the fiber area as the reference space Capillary-to-fiber perimeter exchange index (CFPE) was estimated from the capillary-to-fiber surface area Fiber cross-sectional area (FCSA) and perimeter (FP) was measured with the image analysis system and commercial software (SigmaScan Pro v. 5.0, Systat Software, Inc., Point Richmond, CA, USA). This approach is routinely used in our laboratory (Hüttemann et al., 2012, 2013; Lee et al., 2012; Malek et al., 2013).

### PROTEIN ANALYSIS

The Western blot procedure was consistent with our previous work and detailed elsewhere (Hüttemann et al., 2012, 2013; Lee et al., 2012; Malek et al., 2013). Samples were loaded onto 7.5% (TSP-1, PGC-1α, PGC-1β, and ADAMTS-1) or 12% TGX pre-cast gels (Bio-Rad, Hercules, CA, USA).

The mouse monoclonal primary antibodies used were TSP-1 (1:500, sc-59886, Santa Cruz Biotechnology, Inc), CD47 (1:500; 3847-1, Epitomics), PGC-1β (1:100; sc-373771, Santa Cruz Biotechnology, Inc), α-tubulin (1:2,000, ab11304, Abcam), ADAMTS1 (1:500; sc-47726, Santa Cruz Biotechnology, Inc), and GAPDH (1:2,000, ab9484, Abcam). The polyclonal primary antibodies used were VEGF (1:500, sc-507, Santa Cruz Biotechnology, Inc), VEGFR2 (1:500; 2479, Cell Signaling), FoxO1 (1:200; 2880, Santa Cruz Biotechnology, Inc), Anti-TFAM (1:1,000; ab131607, Abcam), and PGC-1α (1:1,000; AB3242, Millipore). The secondary antibodies used were goat anti-mouse IRDye (1:30,000) and goat anti-rabbit IRDye (1:30,000) purchased from Li-Cor Biosciences. Loading control for target proteins were normalized to α-tubulin or GAPDH. Quantification of bands were analyzed with the Odyssey software program (Li-Cor Biosciences).

### CITRATE SYNTHASE AND CYTOCHROME *c* OXIDASE ACTIVITY

Citrate synthase activity was measured in the lateral gastrocnemius muscle of animals in each group. The method of Srere (1969) was used and samples were analyzed with a Beckman DU 730 spectrophotometer (Beckman, Fullerton, CA, USA) at 412 nm. All samples were tested in triplicate and measured at 37°C as we have done before (Hüttemann et al., 2012; Malek et al., 2013).

To determine the acute effects of (–)–epicatechin on the kinetics of cellular respiration independent of the 8 weeks of (–)–epicatechin treatment and/or the endurance training intervention frozen quadriceps muscles from animals in the control group were incubated in solution containing 20 μM final concentration of (–)–epicatechin or water (placebo) for 25 min. CcO specific activity was measured with a micro Clark-type oxygen electrode in a closed chamber (Oxygraph system, Hansatech, Norfolk, England) at 25°C. Frozen cells were solubilized in 10 mM K-HEPES (pH 7.4), 40 mM KCl, 1% Tween 20, 2 μM oligomycin, 1 mM PMSF, 10 mM KF, 2 mM EGTA, and 1 mM Na vanadate, as described (Lee et al., 2005). CcO activity was measured in the presence of 20 mM ascorbate by addition of increasing amounts of cytochrome *c* from cow heart (Sigma-Aldrich, St. Louis, MO, USA). Oxygen consumption was recorded on a computer and analyzed with the Oxygraph software. Protein concentration was determined with the DC protein assay kit (Bio-rad, Hercules, CA, USA). CcO specific activity was defined as consumed O_2_ (μM)/min/mg total protein.

### STATISTICAL ANALYSIS

Separate one-way ANOVAs were performed to compare differences between group means for each outcome variable. In addition, a separate 4 [group: C, CE, (–)–Epi, and (–)–Epi-Ex] × 3 (time: 0 weeks, 4 weeks, and 8 weeks) mixed factorial ANOVA was performed for body mass. To determine changes in exercise capacity, a separate 4 [group: C, CE, (–)–Epi, and (–)–Epi-Ex] × 2 [time: 0 weeks, and 8 weeks] mixed factorial ANOVA was performed with total distance ran as the dependent variable. To determine the acute effect of (–)–epicatechin on the quadriceps muscle of the control group CcO specific activity was analyzed using a 2 [group: placebo or 20 μM of (–)–epicatechin] × 10 (cytochrome *c* concentration: 0, 1, 2, 3, 5, 10, 15, 20, 25, and 30) mixed factorial ANOVA was conducted. Whenever the overall *F-ratio* was significant *post hoc* Tukey’s *HSD* was performed. Statistical significance was set at *p* ≤ 0.05 for all analyses.

## RESULTS

### ANIMAL BODY AND MUSCLE MASS

The group × time mixed factorial ANOVA for body mass revealed a significant interaction [*F*(6,60) = 2.67; *p* = 0.023]. The follow-up analyses revealed no significant differences between groups (Table 1). There was, however, a significant main effect for time [*F*(2,60) = 10.67; *p* < 0.001]. Therefore, the follow-up analyses indicated that mice were heavier at week 0 (33.0 ± 0.4 g) compared to weeks 4 (31.9 ± 0.4 g) and 8 (32.4 ± 0.4 g). In addition, mice in week 4 weighed significantly (*p* = 0.02) less than mice in week 8.

**Table 1:** **Comparisons of body and muscle masses across the four groups (mean ± SEM)**.

	**Groups**											
	**C**			**(–)–Epi**			**CE**			**(–)–Epi-Ex**		
	**(*n* = 8)**			**(*n* = 9)**			**(*n* = 9)**			**(*n* = 8)**		
	0-weeks	4-weeks	8-weeks	0-weeks	4-weeks	8-weeks	0-weeks	4-weeks	8-weeks	0-weeks	4-weeks	8-weeks
Body mass (g)	33.8 ± 0.9	32.6 ± 0.8	34.0 ± 0.9	31.4 ± 0.8	30.8 ± 0.8	31.7 ± 0.8	33.7 ± 0.8	32.3 ± 0.8	32.1 ± 0.8	33.2 ± 0.9	32.0 ± 0.8	31.7 ± 0.9
Plantaris mass ( mg)	–	–	17.3 ± 0.7	–	–	11.7 ± 0.3^*^	–	–	16.9 ± 0.3	–	–	11.4 ± 0.3^*^
Plantaris/body mass (mg·g^–1^)	–	–	0.51 ± 0.02	–	–	0.37 ± 0.02^*^	–	–	0.53 ± 0.02	–	–	0.36 ± 0.01^*^
Lateral gastrocnemius mass (mg)	–	–	87.0 ± 2.8	–	–	89.1 ± 1.1	–	–	69.6 ± 2.1^**^	–	–	67.5 ± 2.9^**^
Lateral gastrocnemius/body mass (mg·g^–1^)	–	–	2.6 ± 0.1	–	–	2.8 ± 0.1	–	–	2.2 ± 0.1t	–	–	2.1 ± 0.1^***^,^†^
Quadriceps femoris mass (mg)	–	–	213.3 ± 2.1	–	–	191.4 ± 6.1^*^	–	–	212.0 ± 4.5	–	–	205.3 ± 4.1
Quadriceps femoris/body mass (mg·g^–1^)	–	–	6.3 ± 0.2	–	–	6.1 ± 0.2	–	–	6.6 ± 0.2	–	–	6.5 ± 0.1

^*^Significantly (p < 0.001) different from control and CE groups, ^**^significantly (p < 0.001) different from control and (–)–Epi groups, ^***^significantly (p < 0.05) different from control group, ^†^significantly (p ≤ 0.003) different from (–)–Epi group.

The one-way ANOVAs for muscle mass indicated a significant (*p* ≤ 0.007) overall *F*-ratio for the absolute muscle mass for the plantaris, lateral gastrocnemius, and quadriceps femoris as well as the relative muscle masses. As shown in Table 1, the follow-up tests revealed significant mean differences in absolute and relative muscle masses between the four groups.

### ENDURANCE PERFORMANCE

The 4 × 2 mixed factorial ANOVA revealed a significant group × time interaction [*F*(3,30) = 189.35; *p* < 0.001] for total distance ran. We also observed main effects for group [*F*(3,30) = 168.95; *p* < 0.001] and time [*F*(1,30) = 1266.06; *p* < 0.001], but due to the significant interaction these main effects were not interpreted (Keppel and Wickens, 2004). Following the significant interaction, simple main effects test were conducted to determine mean differences among the four groups. At 8 weeks these analyses indicated a statistically significant (*p* < 0.001) increase in total running distance for the (–)–Epi (226.0 ± 2.8 m), CE (194.7 ± 2.8 m), and (–)–Epi-Ex (247.0 ± 3.0 m) groups compared to the control group (134 ± 2.9 m). In addition, we observed the following statistically significant (*p* < 0.001) pattern (–)–Epi-Ex > (–)–Epi > CE for comparisons between the three experimental groups.

### MUSCLE CAPILLARITY

**FIGURE 1:**
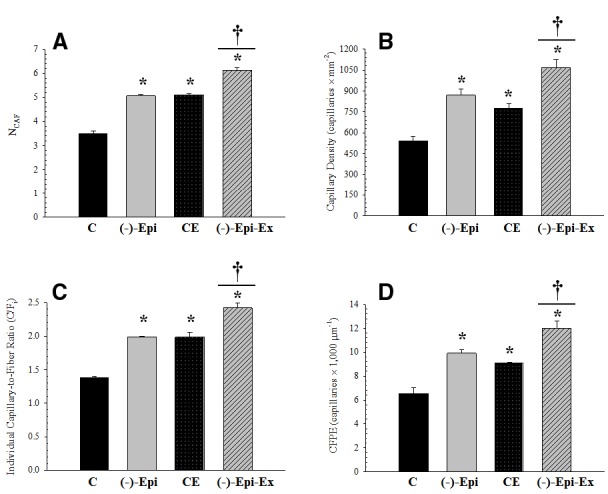
**Measurements of capillarity for the plantaris muscle. (A)** (N*_CAF_*): *significantly different than control group (*p* < 0.0001); ^†^significantly different than Epi and CE groups (*p* < 0.0001). **(B)** (CD): *significantly different than control group (*p* ≤ 0.018); ^†^significantly different than Epi (*p* = 0.043) and CE (*p* = 0.005) groups. **(C)** (C/F*_i_*): *significantly different than control group (*p* < 0.001); ^†^significantly different than Epi and CE groups (*p* = 0.001). **(D)** (CFPE): *significantly different than control group (*p* ≤ 0.013); ^†^significantly different than Epi (*p* = 0.037) and CE (*p* = 0.006) groups. (*n* = 3 per group; mean ± SEM).

The results of the one-way ANOVAs for the various capillary indices revealed significant mean differences among the four groups (Figure 1). Morphometric analyses, however, indicated no significant differences in mean fiber area and perimeter across the four groups (Table 2).

**Table 2:** **Morphometric analyses of the plantaris muscle (mean ± SEM)**.

	**Groups**			
	**C**	**(–)–Epi**	**CE**	**(–)–Epi-Ex**
Plantaris muscle				
Area (μm)	2645 ± 125	2375 ± 98	2667 ± 219	2337 ± 98
Perimeter (μm)	215 ± 14	203 ± 5	221 ± 9	204 ± 7

No significant differences between groups for either index.

### PROTEIN EXPRESSION FOR ANGIOGENESIS

**FIGURE 2:**
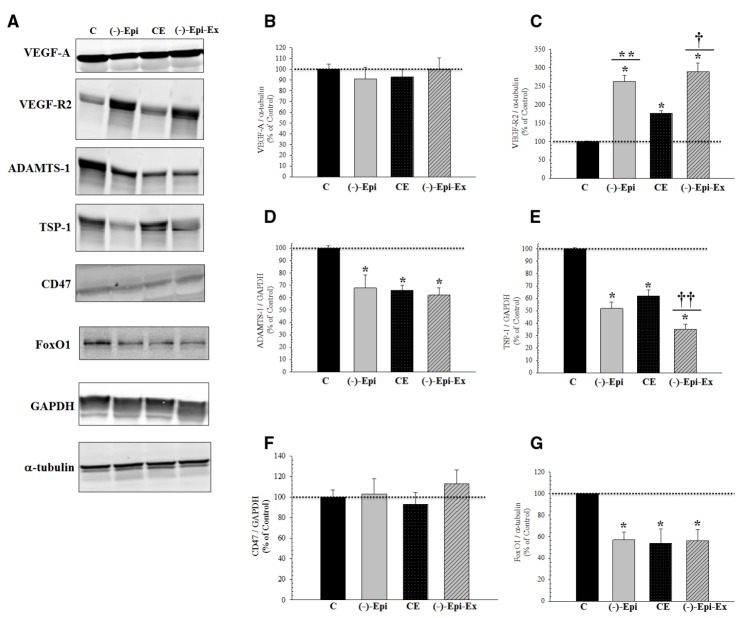
**Basal pro- and anti-angiogenic responses in the quadriceps femoris muscle following 8 weeks of endurance training with and without (–)–epicatechin. (A)** are representative Western blots. **(B)** (VEGF-A) there was no significant difference between groups. **(C)** (VEGF-R2): *significantly (*p* < 0.03) different than control; **significantly (*p* = 0.018) different than CE; and ^†^significantly (*p* = 0.002) different than CE. **(D)** (ADAMTS-1): *significantly (*p* ≤ 0.03) different than control. **(E)** (TSP-1): *significantly (*p* < 0.001) different than control; ^††^significantly (*p* < 0.04) different than (–)–Epi and CE groups. **(F)** (CD47) there was no different between groups for TSP-1 receptor. **(G)** (FOXO1): *significantly (*p* < 0.04) different than control. (*n* = 4–5 animals per group; mean ± SEM).

The results of the one-way ANOVAs revealed significant mean differences in protein expression for regulators of angiogenesis (Figure 2). VEGF-R2 was significantly increased whereas ADAMTS1, TSP-1, and FoxO1 were significantly decreased in the three experimental groups. Moreover, we found no change in protein levels for TSP-1 receptor, CD47.

### PROTEIN EXPRESSION FOR MITOCHONDRIAL PROLIFERATION

**FIGURE 3:**
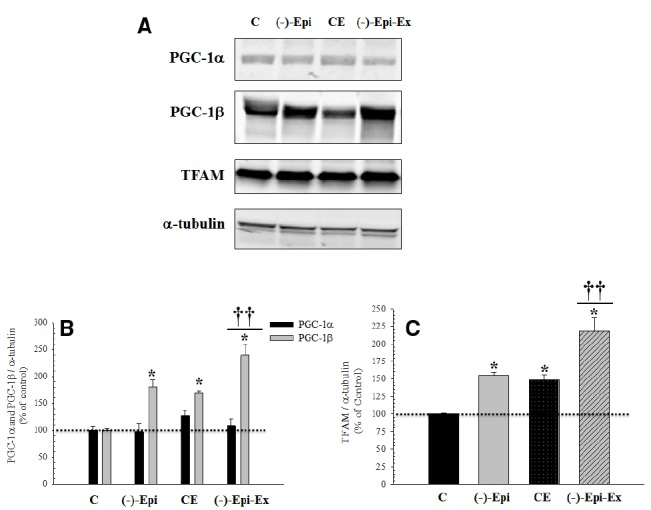
**Basal protein expression of mitochondrial biogenesis regulators in the quadriceps femoris muscle following 8 weeks of endurance training with and without (–)–epicatechin. (A)** are representative Western blots. **(B)** (PGC-1α and PGC-1β): no significant differences between groups for PGC-1α, whereas for PGC-1β *significantly (*p* ≤ 0.014) different than control group; ^††^significantly (*p* ≤ 0.02) different than (–)–Epi and CE groups. **(C)** (TFAM): *significantly (*p* < 0.04) different than control group; and ^††^significantly (*p* ≤ 0.015) different than (–)–Epi and CE groups. (*n* = 4–5 animals per group; mean ± SEM).

As shown in Figure 3, there were significant changes in protein expression for regulators of mitochondrial biogenesis, PGC-1β and TFAM, as a result of endurance training, (–)–epicatechin treatment, or both. Importantly, in the majority of cases, the group receiving both (–)–epicatechin and endurance training had higher protein expression for indices of mitochondrial biogenesis than the groups receiving (–)–epicatechin or endurance training.

### CITRATE SYNTHASE AND CcO SPECIFIC ACTIVITY

**FIGURE 4:**
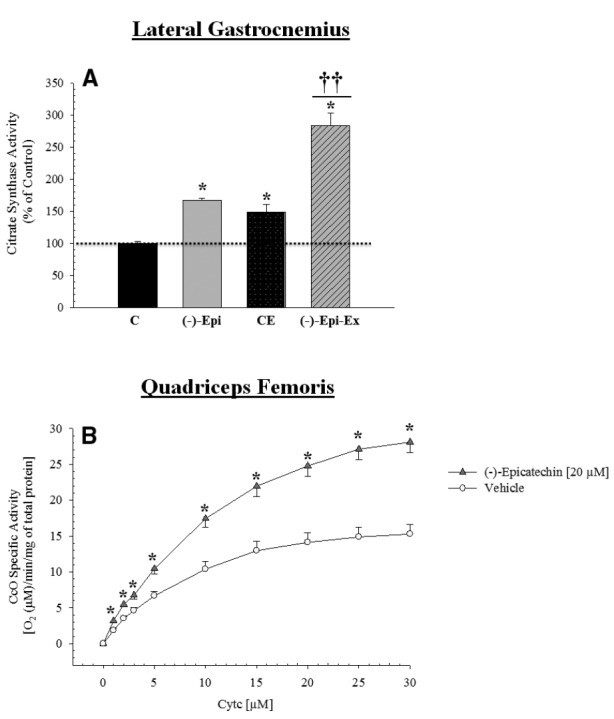
**Citrate synthase and cytochrome c oxidase activity is increased in the hindlimb muscles after (–)–epicatechin treatment. (A)** is citrate synthase activity in the lateral gastrocnemius muscle normalized to the control group *significantly different than control [*p*-values ranged from ≤ 0.007 to 0.048; ^††^different than (–)–Epi and CE, *p* < 0.0001; *n* = 4 animals per group]. **(B)** is CcO activity which was determined in the muscle of mice in the control group incubated in the presence (20 μM final concentration; triangle) or absence (vehicle, circle) of (–)–epicatechin for 25 min using the polarographic method by increasing the amount of substrate cytochrome *c*. CcO activity is defined as consumed [O_2_ (μM)/min/protein mg] (*p ≤ 0.001 significantly different from placebo group, *n* = 4–5 animals per group, mean ± SEM).

Citrate synthase activity increased significantly in all three groups compared to the control group (Figure 4A). Moreover, the (–)–Epi–Ex group had a higher value than the two other experimental groups. In addition, a group × cytochrome *c* concentration mixed factorial ANOVA revealed a significant interaction [*F*(9,63) = 31.85, *p* < 0.0001] for CcO activity measured in the quadriceps femoris muscle. As shown in Figure 4B, the follow-up analyses indicated that for all substrate cytochrome *c* concentrations other than zero, there was a significant increase in mitochondrial respiration in the muscles incubated in 20 μM (–)–epicatechin for 25 min compared to the vehicle treated muscle.

## DISCUSSION

The main finding of the current study was that mice in the group that received (–)–epicatechin treatment combined with 8 weeks of endurance training had increases in some regulators of angiogenesis and mitochondrial biogenesis. This cumulative effect in signaling resulted in increased capillarity and oxidative capacity. From a functional perspective, animals in this group had the highest exercise tolerance than groups receiving either (–)–epicatechin treatment or exercise.

### SKELETAL MUSCLE CAPILLARITY WITH ENDURANCE TRAINING AND (–)–EPICATECHIN TREATMENT

Previous studies have examined the role of (–)–epicatechin on angiogenesis in the brain (van Praag et al., 2007) and skeletal muscle (Nogueira et al., 2011; Hüttemann et al., 2012, 2013). For example, (–)–epicatechin treatment and endurance exercise revealed an increase in cognitive function in mice (van Praag et al., 2007). Nogueira et al. (2011) reported increased capillarity with (–)–epicatechin treatment and light exercise in the plantaris muscle. Both studies, however, did not examine regulators of angiogenesis (i.e., VEGF-A and TSP-1). Olfert and Birot (2011) have suggested that capillary development and/or maintenance may be due to an interaction between pro- and anti-angiogenic growth factors. Hüttemann et al. (2012, 2013) were the initial studies to report changes in pro- and anti-angiogenic regulators with (–)–epicatechin treatment.

In the current investigation we examined the angiogenic response to (–)–epicatechin treatment in conjunction with endurance training. We found that compared to the control group, capillarity increased similarly for groups receiving either (–)–epicatechin or exercise (range 39–60%). The combination of (–)–epicatechin treatment with endurance training, however, increased capillarity (range 74–97%) compared to the control group. The increase observed in the (–)–Epi-Ex group also represented an additional increase (range 32–40%) compared to the (–)–epicatechin or exercise groups. To determine the mechanism associated with the increase in capillarity we examined protein expression of recognized pro- and anti-angiogenic factors (Figure 2). Although we found that basal VEGF-A expression did not change in any of the experimental groups there was a significant increase in its receptor (VEGF-R2). Exercise-induced increase in VEGF-R2 has been reported previously in young, healthy mice following 8 weeks of endurance training (Malek et al., 2013), whereas the lack of increase in basal VEGF-A protein expression following endurance training is consistent with previous studies (Malek et al., 2010). Hüttemann et al. (2013) reported that (–)–epicatechin increased basal VEGF-A protein expression in the plantaris muscle of rats bred for innate LCR. This may suggest that the (–)–epicatechin-induced VEGF-A expression occurs under conditions in which the muscle is compromised such as severe deconditioning (Hüttemann et al., 2013).

Studies reporting increases in basal VEGF-A protein expression in the hindlimb muscles have used voluntary wheel running as their mode of endurance training (Waters et al., 2004; Zwetsloot et al., 2008; Olenich et al., 2013; Ringholm et al., 2013). Therefore, the duration of exercise per day for each animal is significantly higher compared to treadmill exercise. Olenich et al (Olenich et al., 2013) reported that the average exercise time mice spent on the running wheel ranged from 205 min (∼3.4 h) to 363 min (∼6.1 h). Similarly, Allen et al. (Allen et al., 2001) reported an average of 4.3 h of nightly wheel-running activity in mice. In the present study the exercise session was 1 h per day Monday through Friday for a total of 5 h each week. Thus the lack of an increase in basal VEGF-A protein expression in the current study may be explained by the relatively low volume of exercise when using treadmill exercise versus voluntary wheel running.

When anti-angiogenic factors were observed, we found that basal ADAMTS-1 protein expression significantly decreased in all three experimental conditions compared to the control group. Furthermore, we found that basal TSP-1 protein expression was significantly lower in the (–)–Epi-Ex group compared to the three other groups (Figure 2). Basal TSP-1 protein expression in the quadriceps muscle was significantly reduced by either (–)–epicatechin treatment (48%) or endurance training (38%), corresponding with a significant increase in capillarity compared to the control group. The combination of (–)–epicatechin and endurance training ((–)–Epi-Ex) resulted in an even greater decrease of basal TSP-1 compared to the (–)–Epi and CE groups corresponding to higher induction of capillarity (Figure 1). In the present investigation we did not examine the angiogenic response in cardiac muscle as the focus was mainly on skeletal muscle. Nevertheless, previous studies using (–)–epicatechin treatment have shown increased angiogenesis signaling with (–)–epicatechin treatment (Nogueira et al., 2011; Ramirez-Sanchez et al., 2012). Therefore, the increase in exercise tolerance observed in the (–)–Epi-Ex group may also be, in part, due to potential improvements in cardiac vascularization.

A novel finding in the present study was the effects of (–)–epicatechin on FoxO1 protein expression either alone or in combination with endurance training. Recently, Roudier et al. (2013) reported that FoxO1 regulates TSP-1. In addition, Potente et al. (Potente et al., 2005) reported that silencing of either FoxO1 or FoxO3a significantly increased sprout-forming capacity of endothelial cells. Furthermore, Behl et al. (Behl et al., 2009) showed that inhibition of FoxO1 reduced microvascular cell loss in the retina of diabetic rats, whereas Kim et al. (2011) reported that phosphorylation of FoxO1 had an anti-angiogenic function in gastric carcinoma specimens. Slopack et al. (2014) found that protein expression of FoxO1 and FoxO3a was significantly attenuated after 10 days of endurance training when compared to sedentary controls. This pattern of response in skeletal muscle was also observed for TSP-1 protein expression (Slopack et al., 2014). In addition, Slopack et al. (2014) found that endothelial FoxO null mice had a higher capillary-to-fiber ratio after 7 days of endurance training, whereas this increase in capillarity was observed after 14 days of training in wildtype mice. Here, we found that (–)–epicatechin, endurance exercise, or the combination of both reduced FoxO1 protein expression in the quadriceps muscle relative to the control group. To our knowledge, this is the first study to show significant reductions in FoxO1 with (–)–epicatechin treatment alone or in combination with endurance training. Future studies, however, are needed to identify converging pathways between FoxO1 and TSP-1 as it relates to angiogenesis.

### MITOCHONDRIAL SIGNALING WITH ENDURANCE TRAINING AND (–)–EPICATECHIN TREATMENT

Mitochondria have been called the “powerhouses” of the cell, because they provide energy to the working muscles during activities greater than 3 min (Coburn et al., 2012). Furthermore, the mitochondrion is dynamic and can adapt to external stimuli such as exercise (Hoppeler and Fluck, 2003). Although studies have shown that endurance training stimulates mitochondrial biogenesis and therefore increased energy production the mechanism is still under investigation. Some investigators have focused on the PGC-1 proteins (PGC-1α and PGC-1β) to determine their role in skeletal muscle mitochondrial biogenesis (Ikeda et al., 2006; Brault et al., 2010; Rowe et al., 2011; Li et al., 2011; Derbre et al., 2012). Moreover, it has been hypothesized that activation of PGC-1α may be responsible for the initial phase of mitochondrial adaptation to endurance exercise, whereas the subsequent increase in PGC-1β protein expression facilitates the maintenance of mitochondrial biogenesis in the working muscle (Wright et al., 2007). The subsequent activation of PGC-1 proteins induces expression of mitochondrial transcription factor A (TFAM) which regulates mitochondrial DNA gene expression (Ekstrand et al., 2004).

Although the Nogueira et al. (2011) study did examine TFAM protein expression in the hindlimb muscles, they did not examine PGC-1 and FoxO1 protein expressions, or CcO specific activity following acute exposure to (–)–epicatechin. In the present study, we examined key components involved in mitochondrial regulation. To this extent, we found that basal protein expression of PGC-1α was not significantly different between the four groups, whereas basal protein expression of PGC-1β was significantly different in the three experimental groups compared to the control group. The groups that received either (–)–epicatechin or exercise had similar increases after 8 weeks of training, whereas the combination of (–)–epicatechin treatment with exercise was associated with a cumulative response (Figure 3). A number of studies have shown that an increase in PGC-1α expression corresponds to a decrease in FoxO expression in skeletal muscle (Sandri et al., 2006; Qin et al., 2010; Chung et al., 2013). For example, Qin et al. (2010) reported a moderately strong negative correlation between expression of FoxO1 mRNA and PGC-1α nuclear protein. Furthermore, the investigators suggested that PGC-1α expression may repress FoxO1 expression. Similarly, Sandri et al. (2006) also reported an inverse relationship between PGC-1α and FoxO3 expression, however, Chung et al. (2013) used a cell culture model and reported that FoxO6 represses PGC-1α promoter activity. In the present study, we also found decreased FoxO1 protein expression across our three experimental conditions, despite no change in PGC-1α protein expression from control levels. We did see, however, an increase in PGC-1β which has been suggested to regulate basal mitochondrial biogenesis (Meirhaeghe et al., 2003; Liesa et al., 2008). Therefore, futures studies are needed to examine the patterns of expression under various perturbations between isoforms of PGC-1 and FoxO.

We also examined the basal protein expression of TFAM and citrate synthase activity and found that the (–)–Epi-Ex group had significantly higher levels than the control group as well as the two other experimental groups. Moreover, we wanted to examine the acute effects of (–)–epicatechin treatment on CcO activity independent of endurance training. That is, we wanted to determine if acute exposure of skeletal muscle to (–)–epicatechin would increase CcO activity. Thus, we incubated the quadriceps femoris muscle from the control group *in vitro* with or without (–)–epicatechin for 25 min. As shown in Figure 4B, muscles incubated with (–)–epicatechin have significantly higher CcO activity than muscles incubated without (–)–epicatechin. This observed increase cannot be accounted for by an increase in protein expression given the short duration and, therefore may be the result of changes in CcO phosphorylation, which can have a decisive effect on CcO activity and thus mitochondrial function (Acin-Perez et al., 2003, 2011; Lee et al., 2005; Prabu et al., 2006; Samavati et al., 2008). The results of the current study are consistent with those of Hüttemann et al. (2013) who also reported that CcO specific activity increased in healthy rat plantaris muscle incubated in (–)–epicatechin (20 μM) for 25 min. Interestingly, when the plantaris muscle from rats with innate LCR were incubated in (–)–epicatechin there was no increase in CcO specific activity. This may suggest potential impairment in signaling pathways controlling the phosphorylation state of CcO. Future studies, therefore, are needed to elucidate the underlying mechanism of increased oxygen uptake seen in muscle after a single 25 min treatment with (–)–epicatechin.

In summary, the results of the present investigation indicate that (–)–epicatechin treatment combined with 8 weeks of endurance training provide a cumulative effect on a number of angiogenic and mitochondrial signaling. An interesting finding was the role of FoxO1 protein expression in both signaling pathways. Nevertheless, these changes in the hindlimb muscles were associated with an enhanced exercise tolerance above and beyond (–)–epicatechin treatment or exercise alone. Moreover, we found that acute treatment of skeletal muscle with (–)–epicatechin significantly increases mitochondrial respiration independent of exercise. These findings in conjunction with our previous studies (Nogueira et al., 2011; Hüttemann et al., 2012, 2013) on (–)–epicatechin suggest a potential therapeutic effect to enhance capillarity and mitochondrial function either with or without endurance exercise.

## AUTHOR CONTRIBUTIONS

Conception and design of study were by MHM. All authors contributed to collection, analysis and interpretation of data and drafting the manuscript. All authors approved the final version of the manuscript for publication.

### Conflict of Interest Statement

The authors declare that the research was conducted in the absence of any commercial or financial relationships that could be construed as a potential conflict of interest.

## Abbreviations

ADAMTS-1: disintegrin and metalloproteinase with thrombospondin motifs 1
ANOVA: analysis of variance
C: control (C)
CcO: cytochrome *c* oxidase
CE: control with endurance training
(–)–Epi: (–)–epicatechin only
(–)–Epi-Ex: (–)–epicatechin with endurance training
FoxO1: forkhead box O1
PGC-1: peroxisome proliferator-activated receptor gamma coactivator 1
VEGF-A: vascular endothelial growth factor
VEGF-R2: VEGF receptor 2
TSP-1: thrombospondin-1.
